# A coronary anatomy–guided myocardial infarction strategy for optimizing survival and ventricular tachycardia inducibility in a porcine model

**DOI:** 10.1093/europace/euag176

**Published:** 2026-07-11

**Authors:** Juan Mundisugih, Sul Ki Kim, Tejas Deshmukh, Dinesh Selvakumar, Samual Turnbull, Timothy G Campbell, Mitchell Cowan, Michael A Barry, Juntang Lu, Vu Toan Tran, Alan D Marcus, Dhanya Ravindran, Saurabh Kumar, James J Chong, Eddy Kizana

**Affiliations:** Centre for Heart Research, Westmead Institute for Medical Research, Westmead, NSW, Australia; Department of Cardiology, Westmead Hospital, Westmead, NSW, Australia; Sydney Medical School, The University of Sydney, Sydney, NSW, Australia; Centre for Heart Research, Westmead Institute for Medical Research, Westmead, NSW, Australia; Department of Cardiology, Westmead Hospital, Westmead, NSW, Australia; Sydney Medical School, The University of Sydney, Sydney, NSW, Australia; Centre for Heart Research, Westmead Institute for Medical Research, Westmead, NSW, Australia; Department of Cardiology, Westmead Hospital, Westmead, NSW, Australia; Sydney Medical School, The University of Sydney, Sydney, NSW, Australia; Centre for Heart Research, Westmead Institute for Medical Research, Westmead, NSW, Australia; Department of Cardiology, Westmead Hospital, Westmead, NSW, Australia; Sydney Medical School, The University of Sydney, Sydney, NSW, Australia; Department of Cardiology, Westmead Hospital, Westmead, NSW, Australia; Sydney Medical School, The University of Sydney, Sydney, NSW, Australia; Sydney Medical School, The University of Sydney, Sydney, NSW, Australia; Sydney Medical School, The University of Sydney, Sydney, NSW, Australia; Department of Cardiology, Westmead Hospital, Westmead, NSW, Australia; Department of Cardiology, Westmead Hospital, Westmead, NSW, Australia; Department of Cardiology, Westmead Hospital, Westmead, NSW, Australia; Centre for Heart Research, Westmead Institute for Medical Research, Westmead, NSW, Australia; Centre for Heart Research, Westmead Institute for Medical Research, Westmead, NSW, Australia; Sydney Medical School, The University of Sydney, Sydney, NSW, Australia; Department of Cardiology, Westmead Hospital, Westmead, NSW, Australia; Sydney Medical School, The University of Sydney, Sydney, NSW, Australia; Centre for Heart Research, Westmead Institute for Medical Research, Westmead, NSW, Australia; Department of Cardiology, Westmead Hospital, Westmead, NSW, Australia; Sydney Medical School, The University of Sydney, Sydney, NSW, Australia; Centre for Heart Research, Westmead Institute for Medical Research, Westmead, NSW, Australia; Department of Cardiology, Westmead Hospital, Westmead, NSW, Australia; Sydney Medical School, The University of Sydney, Sydney, NSW, Australia

## Introduction

Anterior infarct porcine models are widely used to study post myocardial infarction (MI) ventricular tachycardia (VT); yet, conventional diagonal branch–based infarction strategies typically position the occlusion distal to the second diagonal,^1–3^ assuming similar downstream myocardial territory across animals. This approach does not account for interindividual variation in left coronary anatomy. In clinical populations, such variation influences myocardial perfusion territory, infarct geometry, and border-zone organization. We therefore hypothesized that coronary anatomical variation modulates substrate distribution, acute arrhythmic risk, and VT susceptibility in anterior infarct porcine models. We further sought to develop a probability-based, anatomy-guided infarction strategy to improve acute survival and achieve more consistent VT inducibility.

## Methods

This study comprised a retrospective derivation cohort followed by a prospective feasibility and model-optimization phase. All procedures were approved by the Institutional Animal Ethics Committee and conducted in accordance with national guidelines. The retrospective cohort included 21 mixed-breed female swine (2–4 months, 20–30 kg) undergoing anterior MI and Electrophysiology Study (EPS). In this cohort, animals received prophylactic intravenous amiodarone and lignocaine during infarction. Coronary artery assessment was performed using quantitative coronary angiography with optimal orthogonal projections selected. Animals were categorized as having refractory ventricular arrhythmia (rVA), inducible ventricular tachycardia (iVT), or non-inducible VT (nVT). Anatomical determinants were evaluated using Bayesian multinomial logistic regression (brms, R),^4^ with posterior estimates summarized by 95% credible intervals (CrI). Given the modest sample size, estimates were interpreted cautiously and used mainly to derive posterior probabilities rather than definitive coefficients.

Based on these associations, a probability-based, anatomy-guided occlusion strategy was developed, in which coronary anatomical parameters were used to estimate the probabilities of rVA, and iVT, thereby guiding occlusion-site selection in the prospective cohort to minimize predicted rVA while maximizing predicted iVT. The prospective cohort comprised 20 similarly aged and sized female swine receiving peri-infarction oral sotalol and intravenous lignocaine during MI induction, with sotalol selected for animal welfare considerations, including minimizing the risk of hypotension associated with intravenous amiodarone. An initial exploratory subgroup underwent 90- or 180-min occlusion (*n* = 3 each) to assess infarct-duration effects; subsequent animals (*n* = 14) underwent standardized 90-min occlusion. Serial EPS was conducted at 4 weeks post MI and again 4–10 weeks later. VT inducibility was assessed using programmed stimulation,^5^ with sustained VT and re-entrant mechanisms defined as previously described.^2^ Endocardial electroanatomic (EAM) voltage mapping^6^ and pace-mapping^7^ were performed to characterize substrate, following contemporary principles of VT substrate assessment.^8^

Group comparisons are performed using appropriate statistical testing. In the prospective cohort, logistic regression was performed to identify predictors of inducible VT. Statistical analyses were conducted using R Statistical Software version 4.1.2 (R Foundation, Vienna, Austria), with *P* < 0.05 considered significant.

## Results

**Figure 1:**
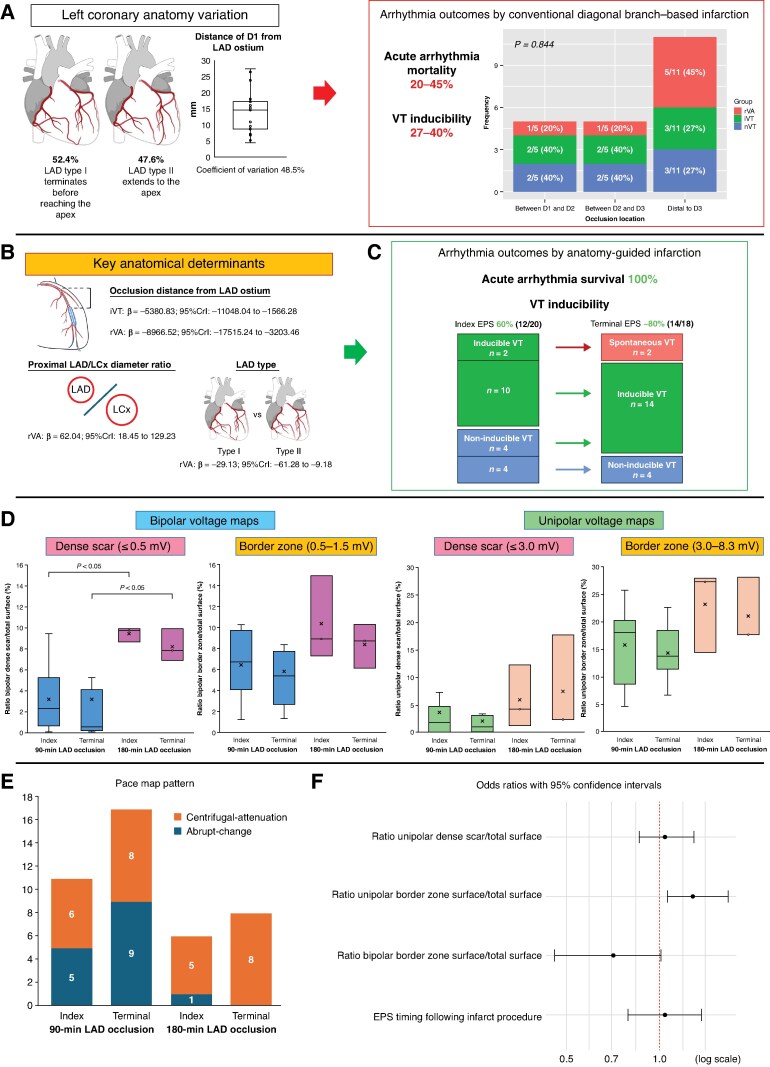
Coronary anatomy shapes substrate distribution and arrhythmia outcomes. (*A*) Substantial variability in left coronary anatomy exists in mixed-breed porcine, particularly in LAD type and ostial-to-D1 distance. Conventional diagonal branch–based infarction strategies were associated with high acute arrhythmia mortality (20–45%) and inconsistent VT inducibility (27–40%), without significant differences in arrhythmia outcomes by occlusion location. (*B*) Posterior estimates indicated that more distal occlusion was associated with lower probabilities of both iVT and rVA. LAD type and the proximal LAD/LCx diameter ratio were primarily associated with rVA, with weaker associations observed for iVT. (*C*) No acute arrhythmia mortality was observed, and VT inducibility increased from 60% at index EPS to approximately 80% at terminal EPS (median follow-up 6 weeks). (*D*) LV endocardial mapping showed marked inter-animal variability in bipolar and unipolar parameters. The 90-min occlusion group had a smaller bipolar dense-scar burden than 180-min occlusion at both index and terminal EPS, while unipolar substrate was similar between groups and unchanged over time. (*E*) The 90-min group showed mixed abrupt-change (AC) and centrifugal-attenuation (CA) patterns, whereas the 180-min group was predominantly CA at index and exclusively CA at terminal EPS. (*F*) Only the unipolar border-zone ratio independently predicted VT inducibility (adjusted OR 1.28; *P* = 0.026), whereas other variables were not significant. The bipolar border-zone ratio showed an inverse trend, consistent with predominant CA-pattern VTs and a deeper substrate contribution.

In the retrospective cohort, substantial variability in left coronary anatomy was observed, particularly in left anterior descending (LAD) type and ostial-to-first diagonal (D1) distance. Across conventional occlusion sites (D1–D2, D2–D3, or distal to D3), acute arrhythmia mortality was 20–45% and VT inducibility 27–40% among survivors (*Figure 1A*). Bayesian multinomial modelling identified associations between coronary anatomical parameters and arrhythmic outcomes. A more proximal occlusion, LAD type 1 and greater proximal LAD/Left Circumflex (LCx) diameter ratio were associated with increased posterior probability of rVA, whereas more distal occlusion locations were associated with lower probabilities of both rVA and iVT, *Figure 1B*.

In the prospective cohort, all animals survived the infarction procedure (100% acute survival). VT inducibility at terminal EPS was achieved in approximately 80% of animals (*Figure 1C*). Ventricular fibrillation was occasionally induced [median 1 (range 0–6) episode/animal at index and 0 (0–2) at terminal EPS] and was managed with cardioversion [median 1 (0–6) and 0 (0–3) shocks/animal, respectively]. Spontaneous VT was identified in 2 animals by implantable telemetry, following clinical deterioration. Inducibility thresholds and reproducibility were not systematically assessed because induction protocols were not repeated after sustained VT for animal welfare reasons.

Among inducible VT animals, EAM revealed marked inter-animal variability (coefficient of variation>20%) in both bipolar and unipolar voltage parameters. Importantly, occlusion duration influenced substrate and VT phenotype. Bipolar dense-scar burden was lower with 90-min occlusion, despite similar unipolar substrate (*Figure 1D*). The 90-min group showed fewer VT morphologies per animal and a higher prevalence of abrupt change patterns, whereas the 180-min group exhibited predominantly centrifugal-attenuation patterns (*Figure 1E*). Among candidate variables, only the unipolar border-zone–to–surface area ratio independently predicted VT inducibility in the prospective cohort (*Figure 1F*).

## Discussion

Left coronary anatomical variation influences acute arrhythmia outcomes and VT inducibility in anterior infarct porcine models. Substantial inter-individual variability in coronary anatomy, particularly in LAD type and ostial-to-D1 distance, was associated with high acute arrhythmia mortality and inconsistent VT inducibility when conventional diagonal branch–based occlusion strategies were used. Within a probabilistic framework, coronary anatomical parameters, including LAD type and proximal LAD/LCx diameter ratio, were associated with arrhythmia outcomes and were incorporated into an anatomy-guided occlusion strategy. No acute arrhythmia mortality was observed in the prospective feasibility cohort, and VT inducibility was high under the anatomy-guided protocol.

Our findings suggest that accounting for coronary anatomical variability may improve outcome consistency of anterior infarct porcine models. Prolonged occlusion increased EAM-derived voltage dense-scar burden without significantly affecting arrhythmia outcomes, whereas a 90-min occlusion was sufficient to achieve high VT inducibility. VT susceptibility in the prospective cohort was more closely related to border-zone distribution than to EAM-derived voltage dense-scar burden alone. These findings suggest that accounting for coronary anatomical variability may reduce experimental outcome variability and improve comparability across preclinical porcine VT studies. Investigators may consider coronary anatomical assessment before infarction to guide occlusion-site selection in mixed-breed porcine VT models.

However, this study has several limitations, including modest sample size, lack of cardiac magnetic resonance imaging for infarct characterization, and potential confounding from differences in anaesthetic and antiarrhythmic protocols. The probabilistic model should be interpreted as hypothesis-generating, and the prospective cohort represents a feasibility evaluation rather than definitive validation.

In conclusion, coronary anatomical variation shapes substrate distribution and influences acute arrhythmia outcomes and VT susceptibility. An anatomy-guided infarct design accounting for biological variability may facilitate more reproducible and comparable preclinical VT studies.

## Data Availability

The data that support the findings of this study are available from the corresponding author, E.K., upon reasonable request.
